# Horses as Sentinels for the Circulation of Flaviviruses in Eastern–Central Germany

**DOI:** 10.3390/v15051108

**Published:** 2023-04-30

**Authors:** Leonard M. R. Gothe, Stefanie Ganzenberg, Ute Ziegler, Anna Obiegala, Katharina L. Lohmann, Michael Sieg, Thomas W. Vahlenkamp, Martin H. Groschup, Uwe Hörügel, Martin Pfeffer

**Affiliations:** 1 Institute of Animal Hygiene and Veterinary Public Health, Faculty of Veterinary Medicine, Leipzig University, 04103 Leipzig, Germany; leonard_gothe@live.de (L.M.R.G.); anna.obiegala@vetmed.uni-leipzig.de (A.O.); 2 Department for Horses, Faculty of Veterinary Medicine, Leipzig University, 04103 Leipzig, Germany; stefanie@ganzenberg.de (S.G.); katharina.lohmann@vetmed.uni-leipzig.de (K.L.L.); 3 Friedrich-Loeffler Institut (FLI), Federal Research Institute for Animal Health, Institute of Novel and Emerging Infectious Diseases, 17493 Greifswald-Insel Riems, Germany; ute.ziegler@fli.de (U.Z.); martin.groschup@fli.de (M.H.G.); 4 Institute of Virology, Faculty of Veterinary Medicine, Leipzig University, 04103 Leipzig, Germany; michael.sieg@mzla.de (M.S.); vahlenkamp@vetmed.uni-leipzig.de (T.W.V.); 5 Animal Diseases Fund Saxony, Horse Health Service, 01099 Dresden, Germany; uwe.hoeruegel@tsk-sachsen.de

**Keywords:** flaviviruses, West Nile virus, horses, seroprevalence, Germany, risk factors, tick-borne encephalitis virus, Usutu virus, virus neutralization test, vector-borne zoonoses

## Abstract

Since 2018, autochthonous West Nile virus (WNV) infections have been regularly reported in eastern–central Germany. While clinically apparent infections in humans and horses are not frequent, seroprevalence studies in horses may allow the tracing of WNV and related flaviviruses transmission, such as tick-borne encephalitis virus (TBEV) and Usutu virus (USUV), and consequently help to estimate the risk of human infections. Hence, the aim of our study was to follow the seropositive ratio against these three viruses in horses in Saxony, Saxony Anhalt, and Brandenburg and to describe their geographic distribution for the year 2021. In early 2022, i.e., before the virus transmission season, sera from 1232 unvaccinated horses were tested using a competitive pan-flavivirus ELISA (cELISA). In order to estimate the true seropositive ratio of infection with WNV, TBEV, and USUV for 2021, positive and equivocal results were confirmed by a virus neutralization test (VNT). In addition, possible risk factors for seropositivity using questionnaires were analyzed using logistic regression based on questionnaires similar to our previous study from 2020. In total, 125 horse sera reacted positive in the cELISA. Based on the VNT, 40 sera showed neutralizing antibodies against WNV, 69 against TBEV, and 5 against USUV. Three sera showed antibodies against more than one virus, and eight were negative based on the VNT. The overall seropositive ratio was 3.3% (95% CI: 2.38–4.40) for WNV, 5.6% (95% CI: 4.44–7.04) for TBEV, and 0.4% (95% CI: 0.14–0.98) for USUV infections. While age and number of horses on the holding were factors predicting TBEV seropositivity, no risk factors were discovered for WNV seropositivity. We conclude that horses are useful sentinels to determine the flavivirus circulation in eastern–central Germany, as long as they are not vaccinated against WNV.

## 1. Introduction

Zoonotic, vector-borne arboviruses from the *Flaviviridae* family, such as West Nile virus (WNV), tick-borne encephalitis virus (TBEV), and Usutu virus (USUV), represent a significant threat to animal and human health. All three are single-stranded RNA viruses with positive polarity [1,2,3,4]. Together with eight other pathogenic flaviviruses, WNV and USUV belong to the Japanese encephalitis serocomplex.

Since the first isolation from the blood of a febrile woman in the West Nile District of Uganda in 1937 [5], WNV has been spread in many countries worldwide, causing minor and more extensive epidemics regularly [6]. Due to genetic variability, genome sequences of the virus can be classified into nine different lineages [7,8]. Lineages 1 and 2 are the most important from a zoonotic point of view and are regularly isolated from birds, humans, and horses during major WNV outbreaks. All lineages replicate in reservoir-competent birds, which are the principal hosts. Birds from the order Passeriformes in particular are considered highly competent amplification hosts with high levels of viremia and massive shedding of virus particles through oral and cloacal fluids [9,10]. Mosquitoes of the genus *Culex* play a significant role as vectors and maintain the infection cycle within bird populations. By also acting as bridge vectors, they transmit WNV to dead-end hosts, such as humans, horses, and other mammals [11,12,13,14]. Compared to birds, the duration and amplitude of viremia in most vertebrate species is insufficient to infect mosquitoes [15].

While WNV lineage 1 is known for causing outbreaks worldwide, lineage 2 was thought to cause WNV infections solely in Africa until 2004 [16,17]. Since 2004, beginning with a Hungarian goshawk fledgling, lineage 2 has also been responsible for WNV outbreaks in southern and eastern Europe [18], and lineage 2 strains have been isolated in Austria (2008), Greece (2010), Romania (2010), and Spain (2017) [19,20,21,22,23,24,25]. The first confirmed WNV isolation in Germany was reported in 2018 after the investigation of homogenized organ material of several dead birds, and these virus isolates also belonged to lineage 2 [24]. Since 2018, WNV infections are endemic in mostly eastern–central Germany, with most cases occurring in Saxony, Saxony Anhalt, and large parts of Brandenburg [26,27,28]. In this area, WNV infections present a considerable threat to endemic horse populations [27].

Although most horses seroconvert without clinical signs, about 8% of infected naive horses [29] develop encephalomyelitis, which is displayed by clinical signs including ataxia, weakness, muscle fasciculations, cranial nerve deficits, and, in severe cases, paralysis and recumbency [30,31]. Three licensed vaccines for horses are available on the European market and provide reliable protection against severe clinical signs [32,33,34,35]. The seroprevalence of WNV infections in the German horse populations has already been investigated in eastern–central Germany, revealing seroprevalence rates of 0% from 2010 to 2012, 8.6% in 2019, and 13.77% in 2020 [36,37] and 5.8% in 2020 [38].

After its first isolation in 1959 in Swaziland, it was long assumed that USUV was restricted to the tropics and subtropics of Africa. However, since its first retrospective appearance in central Europe in 1996 or earlier, the virus has been isolated in several European countries [39,40,41,42]. Due to a very similar transmission cycle, USUV and WNV overlap considerably in terms of their host and vector populations in Europe [43]. Like WNV, USUV is transmitted by mosquitoes, and birds represent the natural reservoir. In particular, blackbirds (*Turdus merula*), great-grey owls (*Strix nebulosa*), and house sparrows (*Passer domesticus*) are considered highly susceptible [44,45]. Similar to WNV, *Culex pipiens* is the most important vector for USUV transmission [46,47]. As with WNV, horses and humans represent accidental hosts because they do not contribute to the transmission cycle after infection [48,49]. While rare cases of human neuroinvasive disease after USUV infection have been described, there are no reports of clinical illness in horses [50,51,52].

TBEV belongs to the tick-borne encephalitis serocomplex [53]. It can be divided into five different genetic subtypes, and it is widely distributed throughout Europe and Asia. In Germany, the European subtype (TBEV-EU) is predominant, and infections are reported mostly from southern Germany [54]. In contrast to WNV and USUV, TBEV is mainly transmitted by hard ticks, and small rodents are the main reservoir [55,56,57]. In Germany, the life cycle of TBEV is maintained by the early developmental stages of castor bean tick (*Ixodes ricinus)* which pick up the virus from reservoir hosts on so-called natural foci [58]. During the next blood meal as nymphs or adults, these ticks infect birds, larger mammals, and humans [59].

Of these three zoonotic flaviviruses, TBEV is of the greatest public health importance since over 10,000 human cases are reported each year in Eurasia [60]. Similar to WNV, a minority of infected human patients suffer from a generalized febrile disease, which can progress to severe illness with mainly neurologic symptoms [61]. Infected horses are often asymptomatic, but cases of febrile and neurologic illness have been described [62,63,64].

Unvaccinated horses are considered a good sentinel species in one-health WNV surveillance systems in southern Europe [65,66], and such studies with horses as the sole early warning system for human infection risk have been reported [67]. In addition to TBEV detection in ticks and the number of reported human cases, serological testing of horses is considered a good tool to assess the abundance of natural TBEV foci in a given area [62,68]. Another advantage of horses as a sentinel species for TBEV is the possibility to reveal microfoci and to perform epidemiological mapping on a smaller geographical scale than the district level [63].

In 2020, a first study investigated the WNV seroprevalence in nine counties in eastern–central Germany and reported risk factors for infection [38]. Due to the continuous geographical spread of WNV, this first follow-up study widens the scope by examining additional counties where cases in birds and/or horses had been newly reported between the autumn of 2020 and the spring of 2022, and by investigating possible seroconversion by retesting WNV-seronegative horses described by Ganzenberg et al. (2022) [38]. We further aimed to evaluate the abundance of TBEV and USUV infection in horses in these counties. Finally, this study repeated and expanded the assessment of potential risk factors for infection with WNV and TBEV on an individual and holding level in order to investigate whether previous results were reproducible.

## 2. Materials and Methods

### 2.1. Study Area and Animals

**Figure 1:**
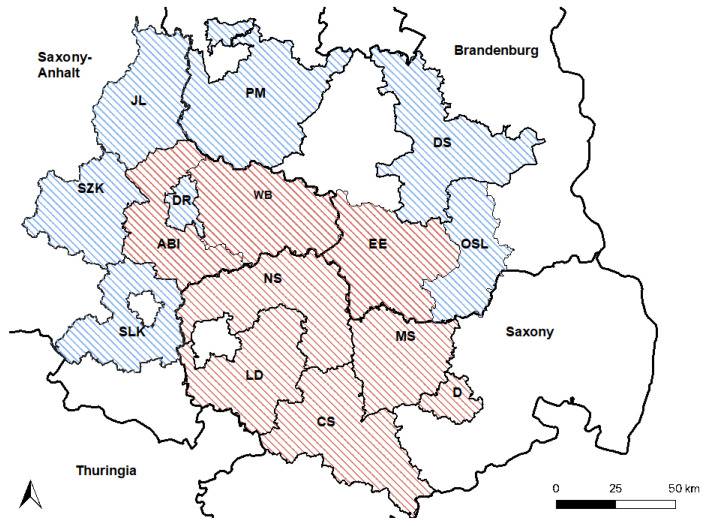
Study area. Counties that were also included in 2020 [38] are shaded in red. Newly included counties are shaded in blue. Saxony-Anhalt: Saalekreis (SLK), Salzlandkreis (SZK), Anhalt-Bitterfeld (ABI), Wittenberg (WB), Dessau-Rosslau (DR), and Jerichower Land (JL). Saxony: Leipzig district (LD), Northern Saxony (NS), Central Saxony (CS), Meissen (MS), and Dresden city (D). Brandenburg: Elbe-Elster (EE), Oberspreewald-Lausitz (OSL), Dahme-Spreewald (DS), and Potsdam-Mittelmark (PM).

The area of investigation was chosen based on the study area reported by Ganzenberg et al. (2022) and was in concordance with officially confirmed cases of WNV infections in horses from eastern–central Germany reported from 2018 to 2021 (Figure 1) [38]. Horses enrolled in the study originated from six counties in Saxony-Anhalt, five counties in Saxony, and four counties in Brandenburg (Figure 1). Compared to Ganzenberg et al. (2022) [38], the study area was extended by adding the counties Salzlandkreis (SZK), Saalelandkreis (SLK), and Jerichower Land (JL) in Saxony-Anhalt, and the counties Dahme-Spreewald (DS), Potsdam-Mittelmark (PM), and Oberspreewald Lausitz (OSL) in Brandenburg (Figure 1). Unfortunately, no holdings in Leipzig city could be included. The study was conducted between January and March of 2022. Of the horses previously tested by Ganzenberg et al. (2022) [38], 219 WNV-seronegative horses were again available for testing. The data of 17 WNV-seropositive horses reported previously [38] were included in an expanded risk factor analyses for WNV-seropositivity. For an expanded risk factor analysis for TBEV-seropositivity, data from 711 horses tested in 2020 [38] were also included.

### 2.2. Epidemiological Data

Horses were recruited by contacting holding managers from all registered holdings with at least five registered equids in the study area. Owners who participated in the earlier study, were approached directly. Due to the low response rate, only a small fraction of the eligible holdings was enrolled in the study. To participate in the study, animals had to be at least one year of age, unvaccinated against the WNV, and kept permanently in the area throughout the year 2021. Since only owners who responded to the call were included, a convenience sample was used in this study.

Information about the horses and the holding was obtained through a standardized questionnaire (File S1, Supplementary Materials) by interviewing the holding manager or the horse owner. In case of contradictory information about the holding, information given by the holding manager was prioritized and applied to all horses living on that holding.

### 2.3. ELISA and VNT

After cleaning and disinfection of the skin, blood samples were collected from the jugular vein of each horse using a sterile vacuum collection system (Vacuette^®^, Greiner Bio-One GmbH, Frickenhausen, Germany). Samples were stored at 4 °C overnight, and serum was separated by centrifugation for 10 min, at 6000× *g* and 10 °C (Heraeus Megarfuge 8R, Fisher Scientific GmbH, Schwerte, Germany). Separated sera were stored at −20 °C until further processing.

All serum samples were tested in duplicate using a commercial panflavivirus competitive enzyme-linked immunosorbent assay (cELISA) (ID-Screen^®^ West Nile Competition Multi-species; IDvet Innovative Diagnostics; Grabels, France), according to the manufacturer’s instructions. This assay is designed to detect specific antibodies to the envelope protein Pr-E of West Nile virus in the sera of horses and various avian species. To confirm or differentiate results of the cELISA, samples with positive or equivocal results were further examined by micro-virus neutralization tests (VNTs), as previously described [69]. In short, sera were incubated at 37 °C for 1 hour with 100 TCID_50_ of a WNV-lineage 2 strain (WNV strain Germany, Gen-Bank accession no. MH924836) after heat inactivation. After the incubation, the sera were added in duplicates to wells with monolayers of target cells. Observable cytopathic effects were recorded one week after infection, and the neutralizing titer (ND_50_) was defined as the reciprocal of the maximum dilution that inhibited cytopathic effects in 50% of the wells. Neutralizing titers of 10 or higher were considered positive. A similar procedure was applied for TBEV (Neudoerfl; GenBank accession no. U27495, Bundeswehr Institute of Microbiology, Munich) and USUV (Europa 3, GenBank accession no. HE599647) [38].

Sera neutralizing more than one virus were considered positive for the virus neutralized at a fourfold higher dilution than all other viruses. If the difference in the dilution was less than fourfold, the sample was considered undifferentiated. Samples that tested positive by cELISA, but negative against all viruses by VNT were considered seronegative. For horses that were included by Ganzenberg et al. (2022) [38] and retested in this study, seroconversion was defined as a positive VNT result in a horse that previously tested negative by VNT in 2020.

### 2.4. Statistical Analysis

The seropositive ratio of each infection on the horse level was calculated as the number of horses testing positive by VNT divided by the total number of tested horses. On the holding level, a seropositive holding was defined as one housing with at least one seropositive horse, and the seropositive ratio was calculated as the number of seropositive holdings divided by the total number of holdings. Within one holding, the seropositive ratio was calculated as the number of seropositive horses divided by the number of participating horses from that holding. The vaccination density of a holding was calculated by the overall horse number and the number of vaccinated horses on the holding.

Analysis of potential risk factors associated with WNV seropositivity was first performed using the chi-squared test. For binomial variables, GraphPad Software (Graph Pad Software InCr., San Diego, CA, USA) was used, while for all other variables, IBM SPSS Statistics version 27.0 (SPSS Inc., Chicago, IL, USA) was used. Continuous variables (age, vaccination density, and number of horses on the holding) were analyzed in Excel via *t*-test (version 2108, Microsoft, Redmond, WA, USA).

To predict the log-likelihood of the outcome, seropositivity against WNV, as an additive function of potential risk factors, a logistic regression model was performed (age, coat color, sex, breed type, country of birth, primary use, primary training location, transport with a distance of more than 20 km from the holding within the last year, clinical signs of neurologic disease in the previous 2 years, clinical signs of febrile disease in the previous two years, travel outside of Germany in the previous two years, location of the holding, number of horses on the holding, type of housing, presence of outdoor shelter, presence of stagnant water within 1 kilometer of the holding, WNV vaccination density on the holding, estimated number of mosquitoes on the holding, use of fly sheets, and additional mosquito control measures). The calculation of effect strength was performed as odds ratios (OR). Results with *p* < 0.05 were considered significant. All variables concerning the logistic regression can be found in the Supplementary Materials (Tables S2 and S3).

In a second analysis, 17 known WNV-positive horses from our previous study [38] that still resided on holdings included in this study, but were not tested again, were included.

The analysis of potential risk factors associated with TBEV seropositivity used six variables (age, number of horses on the holding, type of housing, county, presence of outdoor shelter, and use of repellent) that were expected to influence the TBEV infection risk. Another expanded model was created that included all horses tested for antibodies against TBEV by Ganzenberg et al. [38] and in the current study. Horses that tested seropositive in 2020 and/or seropositive in early 2022 were regarded as seropositive. For twice-tested horses, the most recent information was used in the analyses. The measure of association between significant variables was calculated using Cramer’s V or Pearson correlation coefficient. All analyses of the logistic regression model and Cramer’s V were performed using IBM SPSS Statistics version 27.0 (SPSS Inc., Chicago, IL, USA).

### 2.5. Mapping

Maps were created using the open-source software QGIS (QGIS Geographic Information System, Odense 3.20, Gary E. Sherman et al., Boston, MA, USA). The locations of the holdings were based on Google-derived GPS coordinates (Google Maps, 2021, maps.google.de, accessed on 13 March 2023).

### 2.6. Ethical Statement

The study was ethically approved by the ‘Landesamt für Arbeitsschutz, Verbraucherschutz und Gesundheit Brandenburg’ (Nr. 2347-A-33-1-2020), the ‘Landesverwaltungsamt, Referat Verbraucherschutz, Veterinärangelegenheiten Sachsen-Anhalt (Saxony-Anhalt)’ (AZ: 42502-3- 892KlinikPferd) and the ‘Landesdirektion Sachsen (Saxony)’ (Nr. A06/20). Additionally, horse owners consented in writing before participating in the study.

## 3. Results

Details concerning the selected horse population are presented in Table 1 and Table 2. Of the 1232 horses included in the study, 390 originated from Saxony-Anhalt, 344 from Saxony, and 498 from Brandenburg. A total of 219 horses from 34 holdings that had previously been tested by Ganzenberg et al. [38] and had been seronegative for WNV infection were tested again.

Complete information concerning potential risk factors was obtained for 1211 horses (Table S4). Most horses (n = 1029, 85.1%) were born in Germany. Of those not born in Germany, 51.1% (n = 94) originated from countries with reported WNV cases in the last 10 years. The vaccination density in the study population was 19%. Detailed information about the population characteristics is presented in the Supplementary Materials (Tables S1–S3).

Holdings within the studied area were located between 11°37′ E–13°95′ E and 50°72′ N–52°5′ N and at an altitude between −7 m and 643 m above sea level.

### 3.1. ELISA and VNT

**Figure 2:**
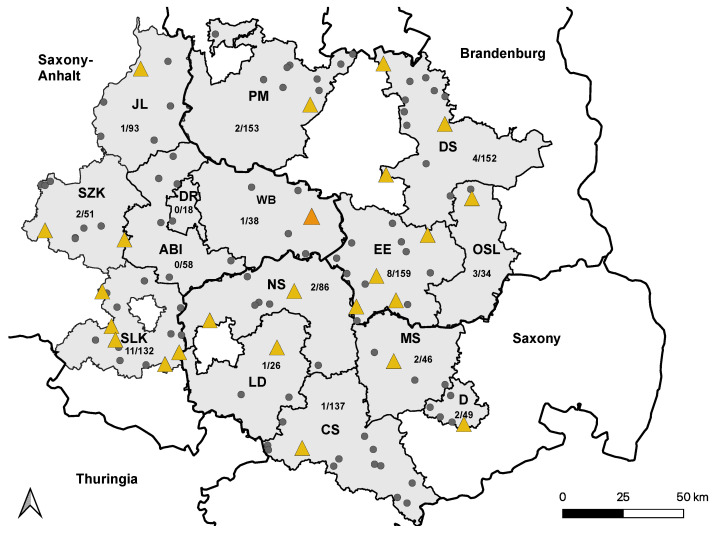
Distribution of WNV-seropositive holdings in 2021. Holdings housing at least one WNV-seropositive horse are marked with yellow triangles. Holdings without WNV-seropositive horses are marked with smaller grey dots. The holding with a single WNV-seroconverted horse are marked with a larger orange triangle. Saxony-Anhalt: Saalekreis (SLK), Salzlandkreis (SZK), Anhalt-Bitterfeld (ABI), Wittenberg (WB), Dessau-Rosslau (DR), and Jerichower Land (JL). Saxony: Leipzig district (LD), Northern Saxony (NS), Central Saxony (CS), Meissen (MS), and Dresden city (D). Brandenburg: Elbe-Elster (EE), Oberspreewald-Lausitz (OSL), Dahme-Spreewald (DS), and Potsdam-Mittelmark (PM).

Of the 1232 horses enrolled in the study, 125 (10.2%) showed flavivirus-specific antibodies based on the cELISA (Table S5). Of those, 40 horses tested positive for neutralizing antibodies against WNV by VNT, with virus-neutralizing titer 50 (ND_50_) ranging from 20 to 1280 (Table 1) and leading to an overall seropositive ratio of 3.3% (95% CI: 2.38–4.40). In total, 5 of the 40 WNV-seropositive horses were born outside of Germany and originated from Spain, Hungary, the Czech Republic, Iceland, and Austria. Additionally, 2 WNV-seropositive horses (5%, 95% CI: 0.50–17.39) had shown signs of febrile illness, but no neurological signs in the 2 years prior to sampling. In one horse that tested WNV-negative in 2020, neutralizing antibodies against WNV were detected in 2022 (Figure 2).

Seventeen horses that were seropositive for WNV infection in 2020 were still present on their respective holdings in 2022, but were not tested again. Assuming that they remained seropositive, the seropositive ratio increased to 4.6% (57/1232) (95% CI: 3.53–5.88) when these horses were included.

**Figure 3:**
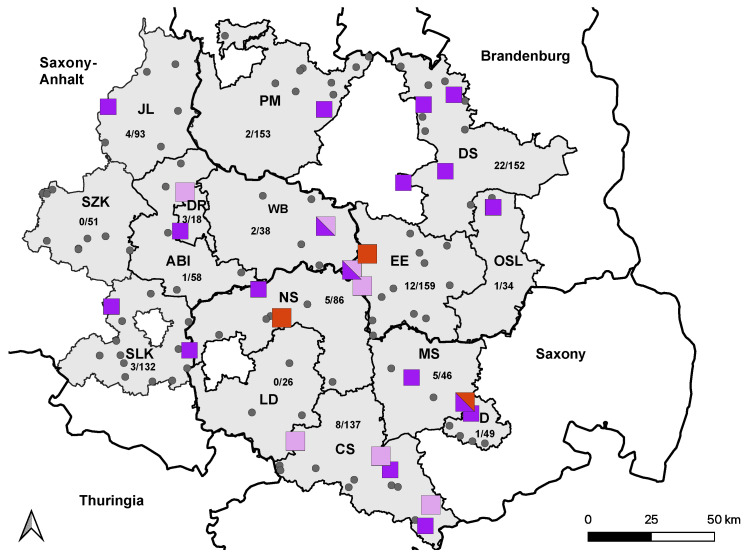
Distribution of TBEV-seropositive holdings in 2021. Holdings housing at least one confirmed TBEV-seropositive horse from 2020 are marked with light-purple squares. Holdings with newly TBEV-seropositive horses from 2021 are marked with dark-purple squares. Holdings without TBEV-positive horses are marked with smaller grey dots. Holdings in which seroconversion occurred in 2021 are marked with bigger red squares. All holdings with horses from more than one category are marked with mixed colors. Saxony-Anhalt: Saalekreis (SLK), Salzlandkreis (SZK), Anhalt-Bitterfeld (ABI), Wittenberg (WB), Dessau-Rosslau (DR), and Jerichower Land (JL). Saxony: Leipzig district (LD), Northern Saxony (NS), Central Saxony (CS), Meissen (MS), and Dresden city (D). Brandenburg: Elbe-Elster (EE), Oberspreewald-Lausitz (OSL), Dahme-Spreewald (DS), and Potsdam-Mittelmark (PM).

Overall, 69 horses were seropositive for TBEV infection, with VNT_50_ titers ranging from 30 to 1920. Of these, 17 horses had already tested seropositive in 2020 [38], and 4 horses seroconverted between 2020 and 2022 (Figure 3). The overall seropositive ratio for TBEV infection, including all horses testing positive by VNT in 2022, was 5.6% (95% CI: 4.44–7.04).

**Figure 4:**
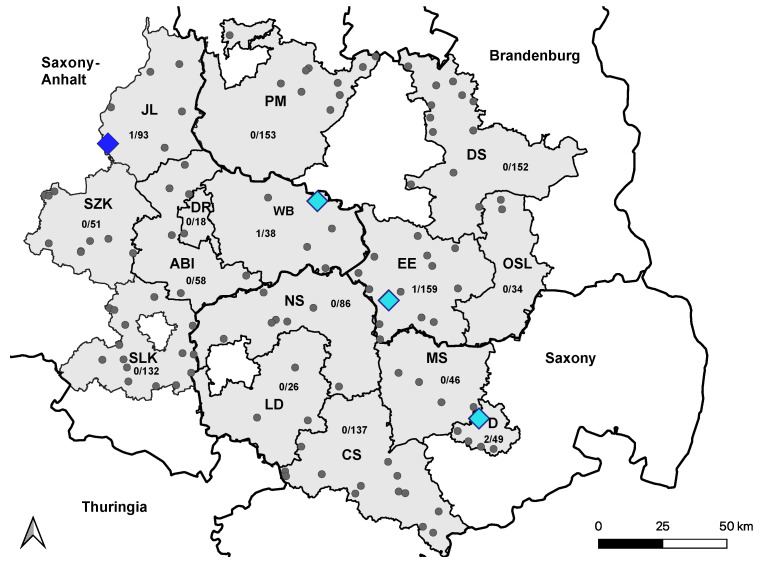
Distribution of USUV-seropositive holdings in 2021. Holdings housing at least one confirmed USUV-seropositive horse from 2020 are marked with light-blue squares. The holding with one newly USUV-seropositive horse from 2021 is marked with a dark-blue square. Holdings without USUV-positive horses are marked with smaller grey dots. Seroconversion to USUV in 2021 was not observed. Saxony Anhalt: Saalekreis (SLK), Salzlandkreis (SZK), Anhalt-Bitterfeld (ABI), Wittenberg (WB), Dessau-Rosslau (DR), and Jerichower Land (JL). Saxony: Leipzig district (LD), Northern Saxony (NS), Central Saxony (CS), Meissen (MS), and Dresden city (D). Brandenburg: Elbe-Elster (EE), Oberspreewald-Lausitz (OSL), Dahme-Spreewald (DS), and Potsdam-Mittelmark (PM).

A total of 5 horses (0.4%; (95% CI: 0.14–0.98) tested positive by VNT for antibodies against USUV, and the ND_50_ titers ranged from 20 to 80. Four of the five horses had previously tested seropositive for USUV infection in 2020 [38], and no seroconversion was observed (Figure 4).

Undifferentiable VNT results were observed in three cases. One horse showed antibodies against WNV and USUV, and two horses against WNV and TBEV. Eight sera that initially tested positive by cELISA showed no neutralizing antibodies in the VNT and were considered seronegative.

The highest seropositive ratio within a single holding was 50% (95% CI: 23.66–76.34) for antibodies against WNV (5/10), 82% (95% CI: 51.14–96.01) for antibodies against TBEV (9/11), and 20% (95% CI: 45.90–52.06) for antibodies against USUV (2/10). In 24% (95% CI: 14.52–29.48) of the holdings tested, at least 1 horse with neutralizing antibodies against WNV was detected. Additionally, 22% (95% CI: 15.26–30.42) of the holdings housed at least 1 horse with neutralizing antibodies against TBEV, and 4% (95% CI: 01.63–10.12) of holdings housed at least 1 horse seropositive for USUV infection (Table 2).

### 3.2. Risk Factor Analysis

No variables with a significant association with WNV seropositivity were found in the univariate analysis. An overview of all variables tested is presented as supplemental data (Tables S6 and S7). The logistic regression model for the outcome of WNV seropositivity was not significant (X^2^ = 6.107; df = 8; *p* = 0.635), resulting in a level of the explainable variance of Nagelkerke’s R^2^ of 0.191.

Of the 22 explanatory variables analyzed in the logistic regression model, none were significant predictors of WNV seropositivity. Complete results are available in Table S7 in the Supplementary Materials.

Six potential risk factors for TBEV seropositivity were entered into the logistic regression analysis (Table S9, Supplementary Materials). The model for the outcome of TBEV seropositivity was nearly significant (X^2^ = 14.696; df = 8; *p* = 0.065), resulting in a level of the explainable variance of Nagelkerke’s R^2^ of 0.037. Of the 6 variables, higher age (OR = 1.034; *p* = 0.032) increased the likelihood, and higher numbers of horses on the holding (OR = 0.991; *p* = 0.031) reduced the likelihood of TBEV-seropositivity. A weak negative correlation between the 2 variables was detected (Cramer-V = 0.203, *p* < 0.001). Younger horses were more likely to be housed on larger holdings. Complete results are available in Table S9 in the Supplementary Materials.

The results of the expanded logistic regression models for WNV and TBEV are presented in the Supplementary Materials (Tables S8 and S10). The results of the expanded model for WNV showed a reduced likelihood of being seropositive in horses housed permanently outdoors (OR = −1.524; *p* = 0.022) and those with reported repellent use (OR = −3.339; *p* = 0.012).

## 4. Discussion

Overall, the seropositive ratio of WNV infection of 3.3% (95% CI: 2.38–4.40) on the horse level and 21% (95% CI: 14.52–29.48) on the holding level is similar to the findings of Ganzenberg et al. [38], where a seroprevalence of 5.8% on the horse level and 21% on the holding level was reported. Since 17 known WNV-positive horses were excluded from sampling, the seropositive ratio determined in this study may be underestimated. On the other hand, an ecological situation more conducive to WNV transmission on twice-tested holdings may have led to an overestimation of the seropositive ratio. Assuming that these 17 horses were still positive during the sampling period, the corrected seropositive ratio would be 4.6% (95% CI: 3.53–5.88) on the horse level and 25% (95% CI: 18.30–34.18) on the holding level.

A recent study on hospitalized horses in Germany reported a seroprevalence of WNV infection of 8.2% (in 2020) and 13.8% (in 2021) [36]. The seroprevalence determined in this study is considerably lower, possibly due to the different study designs, namely, the exclusive investigation of hospitalized horses in the former study [36].

The presented findings are comparable to previous reports from Austria (5.3%) [70], Croatia (3.4%) [71], Greece (4.0%) [72], and northern Spain (5.0%) [73]. Higher seroprevalences ranging from 7.1% to 22.2% were reported in Kosovo, Ukraine, Poland, Corsica, Serbia, and Albania [74,75,76,77,78,79,80,81,82]. Low seroprevalences were reported in Croatia (0.4%) and Poland (0.3%), while no WNV infections were evident in England [83,84,85]. Since WNV has been endemic in southeastern Europe for many years, it is no surprise that a longer period of virus circulation results in higher seroprevalences in these areas. Former studies from Poland and Croatia reporting lower seroprevalences in the past have been updated through recent studies, with higher prevalences showing the ongoing spread of WNV in the equine population in these countries. A similar effect might be expected for Germany, as well. The missing of any confirmed positive results from England is a strong indicator that WNV is not yet present in Great Britain. Since, in the present study, a convenience sample was taken, and former WNV-seropositive equids were excluded from the study population, the comparison of the presented data with other studies is difficult.

The lack of reported neurologic signs is in line with the results of previous studies [29,86,87]. Since none of the horses showed acute clinical signs, and because of the season with a low vector abundance, in which the serum samples were collected, the probability of detecting WNV IgM antibodies or specific WNV viral RNA in the blood was considered very low, and testing was not pursued. To the authors’ knowledge, no acute WNV infections have been reported during the winter season in horses in Germany.

Similar to Ganzenberg et al. [38], eight sera tested positive in the cELISA, but were not confirmed in the neutralization test. Possible explanations include the presence of cross-reacting antibodies against other flaviviruses, for which no neutralization test was performed, such as Japanese encephalitis virus (JEV), Louping ill virus (LIV), or another unrecognized flavivirus; however, JEV and LIV have not yet been proven for Germany [88]. The possibility of early-stage infections without the presence of neutralizing antibodies against WNV, as discussed in Ganzenberg et al. (2022), is considered unlikely in this study, as horses were sampled with more temporal distance to the previous WNV season (January–March vs. September–November). Infections during the season in which sampling took place were considered unlikely due to low temperatures, resulting in a low vector abundance [89].

Since no risk factors for WNV seropositivity were found in the logistic regression, an expanded regression model, including 17 known seropositive horses from the 2020 study, was performed [38]. It revealed a reduced risk for horses permanently kept outdoors and for horses on which a repellent was used (Table S8, Supplementary Materials). The preventive factor of permanent outdoor housing is in contradiction to the previous findings of our group, and a plausible explanation is lacking. The use of a repellent as a protective factor against WNV seropositivity is plausible due to the mosquito bite-preventing effect of these products. Since the significance of both models is low, further investigation is needed to confirm these risk factors.

Our findings showed a seropositive ratio of TBEV infection of 5.6%. Comparable seroprevalence rates were found in Spain (3.1%) and Hesse, Germany (2.9%) [90,91], while higher prevalences were found in Austria (26.1%), Lithuania (37.5%), and Baden-Wurttemberg, Germany (23.4%) [63,92,93]. The seropositive ratio reported here was higher than in the previous study examining the studied area (3.7%) [38]. A possible explanation for this increase in the seroprevalence of TBEV is the inclusion of the county Dahme-Spreewald, which showed the highest seropositive ratio rate of 15% (95% CI: 9.69–21.01). Since holdings with already known high TBEV-seropositive ratios were included, the seropositive ratio of TBEV is likely overestimated.

Age and the number of horses on the holding were risk factors for infection with TBEV (Table S9, Supplementary Materials). Horses with an increased age had a higher risk of being seropositive. These results are in contrast to the findings of a previous study [92], in which a higher proportion of younger horses (mean age 5.9 years) had antibodies against TBEV in a single herd. However, other studies did not find significant associations between age and seropositivity [63]. Our findings agree with previous findings in cattle from Hungary, in which individuals younger than 36 months had a significantly lower seroprevalence rate for infection with TBEV [94]. Due to the observation that all previously TBEV-positive horses still showed TBEV-neutralizing antibodies up to 19 months after the initial testing, a cumulative increase in the rate of seropositivity for TBEV infection with age in the horse population appears likely [38,62].

Another predictor for TBEV seropositivity in this study was the “number of horses on the holding”. Horses on holdings with a higher number of animals were less likely to be seropositive. A possible explanation is the zooprophylaxis theory, in which the presence of cattle in rural malaria areas protects humans from the infection by diverting blood-seeking mosquitoes from human hosts [95]. In accordance with these findings, larger herd sizes could reduce the risk of infection with TBEV by decreasing the likelihood of an infectious tick bite for the individual. Similar effects are discussed in the relationship between black-legged ticks (*Ixodes scapularis*) and white-tailed deer, the latter serving as a possible incompetent dilution host for Lyme disease in North America [96]. Another explanation for the significance of this variable may be the weak correlation with the variable age. This negative correlation is most likely present due to the inclusion of several breeding holdings that are characterized as holdings with high numbers of horses and a high percentage of young horses. These risk factors were not significant in the expanded regression model, which included all previously tested horses from Ganzenberg et al. [38] (Table S10, Supplementary Materials).

Due to the observation that grazing animals are considered a good sentinel for the presence of TBEV in a certain area, one would expect a link between human TBEV cases and the equine seropositive ratio [93,97]. In this study, however, an above-average seropositive ratio of TBEV was detected in counties such as Dahme-Spreewald (15%; 95% CI: 9.69–21.01), which is not registered as an official risk area [98]. On the other hand, some official risk area counties, such as Oberspreewald-Lausitz (3%; 95% CI: 0.01–16.22), showed a below-average seropositive ratio, while others, such as Dessau-Rosslau (17%; 95% CI: 5.01–40.05), showed a high seropositive ratio. These results indicate that TBEV infections in horses happen regularly in the studied area, even if the specific counties still are not recognized as official TBEV risk areas due to the low incidence of human TBEV disease. The fact that three out of four seroconversions occurred in the counties of (1) Elbe-Elster and (2) Northern Saxony supports the theory of active infection cycles in these counties. Therefore, TBEV-seropositive horses could be an additional tool to assess the risk of human TBEV, besides the sole evaluation based on the number of human cases.

Since its first emergence in central Europe in 1996, Usutu virus has been isolated in many European countries, including Germany [40,41]. Although WNV and USUV share many virological aspects in this study, we confirmed previously detected antibodies against USUV in four retested horses and detected one new USUV-seropositive horse, as well as one horse with a potential co-infection of USUV and WNV. Since these horses did not travel abroad and were born in Germany, we conclude that these infections were also acquired autochthonously in the study area. Because holdings with already known USUV-positive equids were included, the seropositive ratio of USUV is likely overestimated.

## 5. Conclusions

This study showed further evidence for the presence of WNV infection in the eastern–central German horse population. The seroconversion in horses for WNV and TBEV demonstrates that these viruses circulate in the study area and were transmitted to horses also in 2021. Nevertheless, horses were WNV sentinels in areas without previous clinical cases in horses and humans. Risk factors predicting seropositivity for TBEV in horses were increasing age and decreasing number of horses on the holding. As searching for TBEV in ticks has been shown to not be constructive, serology in horses is a very useful sentinel tool to detect virus circulation in a given area to detect natural foci. This paper confirmed the presence of equine USUV infections in eastern–central Germany. Finally, this work provided evidence for the presence of neutralizing antibodies against TBEV and USUV in horse sera for at least 15 months in eastern–central Germany.

## Figures and Tables

**Table 1:** Overview of selected horse population and obtained serology results.

Federal State	County	Registered Equids ^#^	Eligible Equids ^⊕^ (in %)	Tested Equids ^◊^ (in %)	WNV-Seropositive Equids ^∇^ (in %)	TBEV-Seropositive Equids ^ᶲ^ (in %)	USUV-Seropositive Equids ^●^ (in %)
Saxony- Anhalt	Saalekreis (SLK)	2507	1444/2507 (58%)	132/1444 (9%)	11/132 (8%)	3/132 (2%)	0/132 (0%)
	Salzlandkreis (SZK)	2347	1044/2347 (45%)	51/1044 (5%)	2/51 (4%)	0/51 (0%)	0/51 (0%)
	Jerichower Land (JL)	2245	1262/2245 (56%)	93/1262 (7%)	1/93 (1%)	4/93 (4%)	1/93 (1%)
	Dessau-Rosslau (DR)	466	285/466 (61%)	18/285 (6%)	0/18 (0%)	3/18 (17%)	0/18 (0%)
	Wittenberg (WB)	1762	857/1762 (49%)	38/857 (4%)	1/38 (3%)	2/38 (*) (5%)	1 */38 (3%)
	Anhalt- Bitterfeld (ABI)	2380	1293/2380 (54%)	58/1293 (5%)	0/58 (0%)	1 */58 (2%)	0/58 (0%)
Saxony	Central Saxony (CS)	4495	1881/4495 (42%)	137/1881 (7%)	1/137 (1%)	8/137 (6 *) (6%)	0/137 (0%)
	Northern Saxony (NS)	3233	1304/3233 (44%)	86/1304 (7%)	2/86 (2%)	5/86 (6%)	0/86 (0%)
	Dresden City (D)	948	428/948 (45%)	49/428 (12%)	2/49 (4%)	1/49 (2%)	2 */49 (4%)
	Meissen (MS)	2749	1189/2749 (43%)	46/1189 (4%)	2/46 (4%)	5/46 (1 *) (11%)	0/46 (0%)
	Leipzig district (LD)	3435	1255/3435 (37%)	26/1255 (2%)	1/26 (4%)	0/26 (0%)	0/26 (0%)
Brandenburg	Elbe-Elster (EE)	2349	1216/2349 (52%)	159/1216 (13%)	8/159 (5%)	12/159 (8 *) (8%)	1 */159 (1%)
	Dahme-Spreewald (DS)	3478	2418/3478 (70%)	152/2418 (6%)	4/152 (3%)	22/152 (15%)	0/152 (0%)
	Oberspreewald- Lausitz (OSL)	1147	620/1147 (54%)	34/620 (6%)	3/34 (9%)	1/34 (3%)	0/34 (0%)
	Potsdam- Mittelmark (PM)	6226	2857/6226 (46%)	153/2857 (5%)	2/153 (1%)	2/153 (1%)	0/153 (0%)
Total		39,767	19,353/39,767 (49%)	1232/19,353 (6%)	40/1232 (3.3%)	69/1232 (17 *) (6%)	5/1232 (4 *) (0.4%)

^#^ Total number of registered equids in respective counties; ^⊕^ Horses housed in holdings with five or more equids were eligible for this study; ^◊^ Eligible equids that were included in this study; ^∇^ Tested equids that were seropositive for WNV; ^ᶲ^ Tested equids that were seropositive for TBEV; **^●^** Tested equids that were seropositive for USUV; * Equids positive in previous testing by Ganzenberg et al. [38] in 2020 and again positive in this study.

**Table 2:** Overview of selected holdings and obtained serology results.

Federal State	County	Registered Holdings ^#^	Eligible Holdings ^⊕^ (in %)	Tested Holdings ^◊^ (in %)	Holdings with ≥1 WNV-Seropositive Horse (in %)	Holdings with ≥1 TBEV-Seropositive Horse (in %)	Holdings with ≥1 USUV-Seropositive Horse (in %)
Saxony- Anhalt	Saalekreis (SLK)	660	106/660 (16%)	15/106 (14%)	5/15 (33%)	2/15 (13%)	0/15 (0%)
	Salzlandkreis (SZK)	760	105/760 (13.8%)	9/105 (9%)	2/9 (22%)	0/9 (0%)	0/9 (0%)
	Jerichower Land (JL)	575	112/575 (20%)	8/112 (7%)	1/8 (13%)	1/8 (13%)	1/8 (13%)
	Dessau-Rosslau (DS)	105	19/105 (18%)	1/19 (5%)	0/1 (0%)	1/1 (100%)	0/1 (0%)
	Wittenberg (WB)	525	80/525 (15%)	4/80 (5%)	1/4 (25%)	1 */4 (25%)	1 */4 (25%)
	Anhalt- Bitterfeld (ABI)	640	109/640 (17%)	6/109 (6%)	0/6 (0%)	1 */6 (17%)	0/6 (0%)
Saxony	Central Saxony (CS)	1662	208/1662 (13%)	14/208 (7%)	1/14 (7%)	5/14 (3*) (36%)	0/14 (0%)
	Northern Saxony (NS)	1256	120/1256 (10%)	7/120 (6%)	2/7 (29%)	2/7 (29%)	0/7 (0%)
	Dresden City (D)	375	42/375 (11%)	5/42 (12%)	1/5 (20%)	2/5 (40%)	1 */5 (20%)
	Meissen (MS)	1053	105/1053 (10%)	4/105 (4%)	1/4 (25%)	1 */4 (25%)	0/4 (0%)
	Leipzig district (LD)	1326	132/1326 (10%)	3/132 (2%)	1/3 (33%)	0/3 (0%)	0/3 (0%)
Brandenburg	Elbe-Elster (EE)	663	103/663 (16%)	13/103 (13%)	4/13 (31%)	3/13 (2 *) (23%)	1 */13 (8%)
	Dahme-Spreewald (DS)	630	160/630 (25%)	12/160 (8%)	3/12 (25%)	4/12 (33%)	0/12 (0%)
	Oberspreewald- Lausitz (OSL)	300	47/300 (16%)	3/47 (6%)	1/3 (33%)	1/3 (33%)	0/3 (0%)
	Potsdam- Mittelmark (PM)	942	165/942 (18%)	10/165 (6%)	1/10 (10%)	1/10 (10%)	0/10 (0%)
Total		11,472	1613/11,472 (14%)	114/1613 (7%)	24/114 (21%)	25/114 (8 *) (22%)	5/114 (4 *) (4%)

^#^ Total number of registered holdings in respective counties; ^⊕^ Holdings that housed five or more equids were eligible for this study; ^◊^ Eligible holdings that were included in this study; * Equids seropositive in previous testing by Ganzenberg et al. [38] in 2020 and again seropositive in this study.

## Data Availability

The results data of the study are available in the publication or the Supplementary Materials. Further specific information regarding the datasets analyzed during the study can be obtained from the corresponding authors upon reasonable request.

## Supplementary Materials

The following supporting information can be downloaded at: https://www.mdpi.com/article/10.3390/v15051108/s1, Table S1: Holdings included in the study and results of serological testing (n = 1232); Table S2: Descriptive statistics for equids included in the study, and answers from questionnaire about potential risk factors for WNV-seropositivity (n = 1211); Table S3: Descriptive statistics for horses included in the study, and answers from questionnaire about potential risk factors for TBEV seropositivity (n = 1211); Table S4: Comparison between the datasets of the present study and Ganzenberg et al. (2022); Table S5: Outcome of the virus neutralization tests (VNT) for 117 horses testing positive or equivocal by competitive ELISA (cELISA); Table S6: univariate analyses (chi-square, *t*-test) of potential risk factors for WNV seropositivity in 1211 horses; Table S7: Logistic regression model (complete data) for the relation of potential risk factors with WNV seropositivity in 1211 horses; Table S8: Logistic regression model (complete data) for the relation of potential risk factors with WNV seropositivity in 1228 horses (17 previously positive horses from 2020 included); Table S9: Logistic regression model (complete data) for the relation of risk factors with TBEV seropositivity in 1211 horses; Table S10: Logistic regression model (complete data) for the relation of risk factors with TBEV seropositivity in 1935 horses (complete dataset of all tested horses in 2020 and 2022); File S1: standardized questionnaire for epidemiological data collection.
