# Supplementary material for: Horses as Sentinels for the Circulation of Flaviviruses in Eastern–Central Germany
Source: Viruses. 2023 Apr 30;15(5):1108. doi: 10.3390/v15051108 (PMC10222594; doi:10.3390/v15051108)
Supplement: Supplementary file 1 [file viruses-15-01108-s001.zip › viruses-2323421-File S1.pdf]

Fragebogen zur Seroprävalenz von WNV-Infektionen bei Pferden

Liebe/r Pferdebesitzer/in,

für die Auswertung der Studienergebnisse benötigen wir einige Angaben zu Ihrem Pferd und bitten Sie um die Beantwortung der folgenden Fragen. Darüber hinaus benötigen wir zur Sicherstellung einer direkten Kommunikation, sowie zur Übermittlung der Testergebnisse, Ihre vollständigen Kontaktdaten. Die bereitgestellten Informationen werden streng vertraulich behandelt und sind ausschlaggebend für eine reibungslose Durchführung der Studie. Nähere Informationen zum Datenschutz entnehmen Sie bitte der „Einwilligungs-/Datenschutzerklärung zur Teilnahme an einer wissenschaftlichen Studie“.

Bitte beachten Sie, dass an dieser Studie nur Pferde teilnehmen können, die bisher noch **nicht gegen** das **West-Nil-Virus geimpft** wurden. Bereits geimpfte Pferde können leider nicht in diese Studie aufgenommen werden.

Für Rückfragen stehen wir telefonisch (01783264322) oder natürlich jederzeit per E-Mail unter leonard.gothe@uni-leipzig.de zu Ihrer Verfügung.

Bitte senden Sie uns die Fragbögen zeitnah ausgefüllt zurück oder bringen Sie diese zum Blutentnahmetermin mit.

Vielen Dank für Ihre Beteiligung, Leonard Gothe.

Kontaktdaten:

Name: ____________________________________Vorname: ________________________________

Adresse: ____

Adresse Stall: ____

Telefon: ____

Email: ____

Identifikation Pferd/e: (Name, Geburtsdatum, Signalement, Lebensnummer)

1:____

2:____

3:____

4:____

5:____

6:____

7:____

8:____

Erklärung zur Beantwortung:

Bitte nehmen Sie sich für die Beantwortung ein paar Minuten Zeit. Hierbei gibt es keine Bewertung und kein Richtig oder Falsch. Allein eine ehrliche Beantwortung ermöglicht uns eine korrekte Analyse der gewonnenen Daten. Wählen Sie bitte bei jeder Frage die Antwort, die am besten zu Ihrem Pferd passt.

Kreuzen Sie bitte nur eine Antwortmöglichkeit an. Bei einigen Fragen können Sie unter „Sonstiges“ Alternativen zu den vorgeschlagenen Kategorien angeben. Bei anderen Fragen haben Sie unter „weitere Informationen“ die Möglichkeit, Ihre Antwort zu erklären. Kreuzen Sie hier bitte trotzdem eine der Antworten an.

Wenn Sie den Fragebogen für mehrere Pferde ausfüllen schreiben Sie bitte den Namen des Pferdes oder die Ziffern aus den Kontaktdaten hinter die jeweilig passende Antwort. Sollten Sie bereits in den Kontaktdaten das vollständige Signalement der Pferde angegeben haben, können Sie diese Fragen im Bogen überspringen.

Sollten ab Frage 9 die Angaben für alle von Ihnen im Fragebogen benannten Pferde identisch sein, so ist ein Kreuz pro Frage ausreichend.

Angaben zu Ihrem Pferd/Ihren Pferden:

1. **Bei meinem Pferd handelt es sich um ein:**
   - ☐ Kaltblut- oder Kaltblutmix
   - ☐ Warmblut
   - ☐ Vollblut
   - ☐ Pony/Kleinpferd
   - ☐ Esel/Muli
   - ☐ Sonstiges: ____
2. **Mein Pferd ist ein/e:**
   - ☐ Stute
   - ☐ Hengst
   - ☐ Wallach
3. **Welchem Fellfarb-Typ entspricht Ihr Pferd?**
   - ☐ Dunkel (Rappe/Dunkelbrauner/Brauner)
   - ☐ Fuchs
   - ☐ Hell (Lichtfuchs/Falbe/Palomino)
   - ☐ Sehr hell bis weiß (Schimmel/Cremello)
   - ☐ Mehrfarbig (Schecke/Appaloosa)
   - ☐ Sonstiges: ____
4. **Ist Ihr Pferd in Deutschland gezogen/geboren worden?**
   - ☐ Ja
   - ☐ Nein

   Wenn Nein, wo? ____

5. **Seit wann befindet es sich in Deutschland?**

____

6. **Hat sich Ihr Pferd innerhalb der letzten 2 Jahre außerhalb Deutschlands aufgehalten?**

☐ Ja

☐ Nein

Wenn Ja, Wo? ________________________________ Wann?________________________

7. **Zeigte Ihr Pferd in den letzten 24 Monaten Symptome einer fiebrigen Allgemeinerkrankung?** (mehrere Antworten möglich)

☐ Gestörtes Allgemeinbefinden/Apathie

☐ Fieber

☐ Schwitzen

☐ Leistungsabfall/Bewegungsunlust

☐ Angestrengte Atmung

☐ Sonstiges

Wenn Ja, wann? ____

8. **Zeigte Ihr Pferd in den letzten 24 Monaten Symptome einer neurologischen Erkrankung?** (mehrere Antworten möglich)

☐ Verhaltensauffälligkeiten (Aggression, Depression, Lethargie)

☐ Gleichgewichts- und Bewegungsstörungen (Trippeln, Stolpern, schwankender Gang, Ataxie)

☐ Muskelzittern

☐ Nervenlähmungen (Krämpfe, Lähmungen z.B. im Kopfbereich, Koma)

Wenn Ja, wann? ____

<u>Haltung und Nutzung:</u>

9. **Wie viele Pferde stehen insgesamt im Betrieb?**

____

10. **Wie wird Ihr Pferd in der insektenreichen Zeit (ca. von April bis November) gehalten?**

☐ Stallhaltung (Innenbox/Außenbox) ohne weiteren Auslauf

☐ Stallhaltung mit kontrolliertem Auslauf (weniger als 12h pro Tag)

☐ Stallhaltung mit kontrolliertem Auslauf (mindestens 12h pro Tag)

☐ 24h Auslauf (Laufstall/Offenstall/Weide)

11. **Wie ist der Auslauf Ihres Pferdes beschaffen?**

☐ Paddock (eingezäunter, meist stallnaher, befestigter Auslauf ohne Grasnarbe)

☐ Koppel (eingezäunte, meist naturnahe Weidefläche mit Grasnarbe)

☐ Kombination von Paddock und Koppel (z.B. Offenstall mit Weidezugang)

Weitere Informationen: ____

12. Steht Ihr Pferd gemeinsam mit anderen Pferden im Auslauf?

- ☐ Ja
- ☐ Nein

Wenn Ja, wie viele? ______________________________

13. Besitzt der Auslauf einen Unterstand?

- ☐ Ja
- ☐ Nein

Art des Unterstandes: ______________________________

14. Befinden sich im Umkreis von ca. 1 km zum Auslauf stehende Gewässer? (Entwässerungsgräben, Tümpel…)

- ☐ Ja
- ☐ Nein

Weitere Informationen: ______________________________

15. Ist Ihr Pferd in den letzten 12 Monaten weiter als 20 km transportiert worden?

- ☐ Ja
- ☐ Nein

Wenn Ja, wohin? ______________________________

16. In welcher Form nutzen Sie Ihr Pferd vorrangig?

- ☐ Freizeit
- ☐ Sport (Dressur/Springen/Fahren/Schulpferde/Landwirtschaftliche Nutzung)
- ☐ Zucht/Aufzucht
- ☐ Weidepferd/Beistellpferd
- ☐ Sonstiges: ______________________________

17. Wo wird Ihr Pferd bewegt (z.B. geritten)?

- ☐ Ausschließlich in der Halle/auf dem Platz
- ☐ Vorrangig in der Halle/auf dem Platz
- ☐ Vorrangig im Gelände
- ☐ Ausschließlich im Gelände
- ☐ Mein Pferd wird nicht besonders bewegt

Weitere Informationen: ______________________________

18. Wie viele Pferde im Betrieb sind gegen das West-Nil-Virus geimpft?

- ☐ Keines
- ☐ Einige, Wie viele? ______________________________
- ☐ Alle

Stechmückenvorkommen und -kontrolle in Ihrem Betrieb:

19. **Wie würden Sie die Stechmückensituation in Ihrem Stall beschreiben?**

- ☐ Massenhaft Mücken
- ☐ Viele Mücken
- ☐ Eher wenig Mücken
- ☐ Keine Mücken

20. **Welche Arten der Insektenbekämpfung kommen am Tier zur Anwendung?**

- ☐ Insektenschutzspray
- ☐ Decken
- ☐ Gels/Salben
- ☐ Futterzusätze

21. **Welche Art von Insektenschutzspray nutzen Sie?**

Selbst hergestelltes Spray, Wirkstoff: ____

Gekauftes Spray, Name des Produktes: ____

22. **In welchen Situationen und wie regelmäßig wenden Sie das Spray an?**

	**Immer (mind. 1x/Tag)**	**Meistens (mind. 1x/Woche)**	**Selten (weniger als 1x/Woche)**	**Nie**
**Im Stall/in der Box**	☐	☐	☐	☐
**Beim Reiten**	☐	☐	☐	☐
**Im Auslauf**	☐	☐	☐	☐
**Beim Transport**	☐	☐	☐	☐
**Sonstiges (bitte angeben):** ________________	☐	☐	☐	☐

23. **Nutzen Sie in der Saison eine Fliegendecke bei Ihrem Pferd?**

- ☐ Ja
- ☐ Nein → Bitte springen Sie zu Frage 25.

24. **Welche Art von Fliegendecke nutzen Sie?**

- ☐ Klassische Fliegendecke
- ☐ Decke mit Muster (z.B. Zebrastreifen)
- ☐ Ekzemerdecke (mit Kopf- und Halsteil)
- ☐ Sonstiges: ______________________

25. Welche der folgenden Bekämpfungsmaßnahmen werden in Ihrem Stall gegen Stechinsekten durchgeführt? (Mehrfachantworten möglich)

	**Trifft zu**	**Trifft nicht zu**
Automatische Tränken sind vorhanden	☐ im Stall<br>☐ im Auslauf	☐ im Stall<br>☐ im Auslauf
Eimer und/oder Tröge sind vorhanden	☐ im Stall<br>☐ im Auslauf	☐ im Stall<br>☐ im Auslauf
Kompletter Wechsel von Tränkwasser (mind. 1x wöchentlich)	☐ im Stall<br>☐ im Auslauf	☐ im Stall<br>☐ im Auslauf
Lamellen an Türen sind vorhanden	☐	☐
Fliegengitter an Fenstern sind vorhanden	☐	☐
Bremsenfallen sind vorhanden	☐	☐
Elektr. Insektenfalle/ „Mückengrill“ ist vorhanden	☐	☐
Fliegen-Klebefänger sind vorhanden	☐	☐
Duftstofffalle ist vorhanden	☐	☐
Sonstiges (Bitte angeben, z.B. Schwalben)		

Gibt es weitere Informationen zu Ihrem Pferd, die Sie uns mitteilen möchten und zu denen wir Sie bisher noch nicht befragt haben? Hier ist Platz für Ihre Notiz:

____

____

____

Dürfen wir Sie für eine Teilnahme an weiterführenden Studien zum Thema West-Nil-Virus beim Pferd kontaktieren?

☐ Ja, bitte nutzen Sie hierfür die oben angegebenen Kontaktdaten

☐ Nein, ich habe kein Interesse an einer weiteren Kontaktaufnahme

Sie haben es geschafft. Ich danke Ihnen für Ihre Teilnahme!
